# A Strategy Towards the Valorization of Aloe Vera Rinds to Obtain Crystalline Cellulose: Pretreatment Effects and Elemental Analysis

**DOI:** 10.3390/polym17040553

**Published:** 2025-02-19

**Authors:** Mayra Elizabeth Juárez Méndez, Diana Palma Ramírez, David Salvador García Zaleta, Karen A. Neri Espinoza, Acela López Benítez, Deyanira del Ángel López, Sandra Soledad Morales García, Helen Willcock

**Affiliations:** 1 Department of Chemical and Biochemical Engineering, Tecnológico Nacional de México (TecNM), Ciudat Madero 89460, Tamaulipas, Mexico; d97070689@cdmadero.tecnm.mx; 2 Department of Polymers and Nanomaterials, Unidad Profesional Interdisciplinaria de Ingeniería Campus Hidalgo (UPIIH), Instituto Politécnico Nacional (IPN), San Agustín Tlaxiaca 42162, Hidalgo, Mexico; kneri@ipn.mx (K.A.N.E.); aclopezb@ipn.mx (A.L.B.); 3 División Académica Multidisciplinaria de Jalpa de Méndez, Universidad Juárez Autónoma de Tabasco (UJAT), Villahermosa 86690, Tabasco, Mexico; david.garcia@ujat.mx; 4 Department of Nanostructured Materials, Centro de Investigación en Ciencia Aplicada y Tecnología Avanzada (CICATA) Unidad Altamira, Instituto Politécnico Nacional (IPN), Altamira 89600, Tamaulipas, Mexico; ddelangel@ipn.mx; 5 Department of Pollution Prevention and Control, Centro Mexicano para la Producción más Limpia (CMPL), Instituto Politécnico Nacional (IPN), Mexico City 07340, Mexico; smoralesg@ipn.mx; 6 Department of Materials, Loughborough University, Loughborough LE11 3TT, UK; h.willcock2@lboro.ac.uk

**Keywords:** Aloe Vera rinds, hydrolysis, crystalline nanocellulose, biomass

## Abstract

Although crystalline nanocellulose (CNCs) can be extracted from different resources, the employed pretreatments, which disrupt the inter- and intramolecular physical interactions, depend on the biomass sources. This study aims to valorize Aloe Vera (AV) rinds into cellulose and crystalline nanocellulose (CNC) employing two approaches during hydrolysis: sulfuric acid (CNC_SA_) and citric acid (CNC_CA_) after 30, 60, and 90 min of reaction. The effects of pretreatments and hydrolysis time on the functional groups and hydrogen bonding in biomass are discussed. Crystalline structure (polymorph type), crystallinity, thermal stability, morphology, particle size, and metal presence are also analyzed. A transformation from type I into II polymorph was achieved, where the intermolecular interactions governing cellulose were increased in CNC_SA_ and were almost maintained in CNC_CA_. Properties based on the structure, thermal properties, particle size, and metal presence indicate that the CNC_SA30_ and CNC_CA90_ samples displayed potential application as reinforcement agents for other types of polymers having no more melting points of 160 and 220 °C, respectively.

## 1. Introduction

Environmental pollution has increased the use of non-traditional renewable sources capable of producing biodegradable nanomaterials [1]. In recent decades, the development of biopolymers as replacements for petroleum-based polymers has continued to grow [2]. Globally, biopolymers have garnered significant attention in the market due to their renewability and environmental benefits, particularly in terms of biodegradability [3]. Biopolymers are considered sustainable and biocompatible materials because they are derived from living resources such as plants [4]. Due to their numerous advantages, biopolymers are used in a wide range of applications, including medicine, electronics, biosensors, adsorbents, and energy storage devices [5,6,7]. Biopolymers are biological macromolecules consisting of covalently bonded monomeric units [8,9].

Cellulose is the most abundant natural and renewable resource on Earth [10]. It is composed of bundles of fibrils known as microfibrils. Each individual fibril contains both crystalline and amorphous regions [11]. Within the amorphous regions, several polymorphs can be formed (I, II, III, and IV). Cellulose I adopts two polymorphs, a triclinic structure (Iα) and a monoclinic structure (Iβ), which coexist in different proportions depending on the origin of cellulose. Additionally, cellulose II has a monoclinic structure and is the most relevant stable form [11,12]. After removing the amorphous regions, cellulose nanocrystals, the rod-shaped crystalline components, are obtained [11].

Cellulose nanocrystals can be used as reinforcing materials to develop biodegradable polymer composites [13]. These nanoscale particles offer advantages, such as being biodegradable, renewable, and lightweight as well as exhibiting high strength and hydroxyl surface chemistry [14,15]. For these reasons, it is an excellent candidate for use as a reinforcing agent in the different polymeric matrixes, enhancing the mechanical, thermal, and barrier properties [16]. However, certain characteristics, such as strong inter-particle interactions due to hydrogen bonds, make it difficult to achieve uniform dispersion when particles are incorporated into hydrophobic polymers. As a result, obtaining a well-dispersed system in hydrophobic polymers remains a challenge that requires further investigation [14]. Nowadays, the most promising candidates for preparing crystalline nanocellulose are waste biomass resources or agricultural waste materials [13].

The chemical composition of lignocellulosic (LC) biomass varies depending on the feedstock type [17]. Generally, LC biomass consists of the following lignocellulosic biopolymers: cellulose (35–55 wt%), hemicellulose (20–40 wt%), and lignin (10–25 wt%) [17]. However, isolating these biopolymers is challenging due to the enzymatic hydrolysis of cellulose into sugar and the complex structure of the lignin, which often results in low yields [18]. This process involves two essential steps: (1) pretreatment and (2) oxidative bleaching. The purpose of pretreatment is to preserve cellulose almost intact while reducing hemicellulose and lignin content [19]. Chemical pretreatments with sodium hydroxide (NaOH) or potassium hydroxide (KOH) at concentrations between 8.0 and 10.0% have shown effectiveness in cleaving uronic and acetic esters, as well as a-ether (phenolic) linkages, facilitating the removal and solubilization of hemicellulose in the medium [20,21]. On the other hand, the oxidative bleaching process allows the oxidization of the insoluble lignin compounds to break the aryl ether, carbon–carbon, and β-O-4 bonds [22]. Buendia-Kandia et al. [23] noted that a treatment with sodium chlorite/acetic acid is accessible and cost-effective and enables the removal of up to 60 wt% of lignin. This process enhances the purity of the biomass while minimizing cellulose loss and achieving a high recovery of hemicellulose. Finally, the extraction from this “purified” cellulose is carried out. According to the literature, acid hydrolysis using sulfuric, hydrochloric, phosphoric, and maleic acids can be used for this purpose [24].

Aloe Vera (AV), Aloe barbadensis Miller, is a perennial desert plant similar to the cactus and belongs to the lily (Liliaceae) family [25]. AV is one of the most abundant natural containing biopolymers on Earth [26]. The rind accounts for approximately 20–30% of the weight of the whole plant leaf, while the pulp represents approximately 65–80% [27]. The AV gel is a colorless substance obtained from the leaf pulp and mainly consists of water (>98%) [25,27]. This gel is thought to be responsible for most of the plant’s therapeutic properties [28] and is extensively employed as a material for edible films/coatings [29,30], making it a valuable product in industrial contexts [31]. The AV gel is complex, containing various bioactive compounds, such as carbohydrates, sugars, minerals, organic acids, vitamins, proteins, fibers, and phenolic compounds [32]. The AV rind is the outer thick green layer and it works to protect the gel matrix. It is currently considered agricultural waste [31]. Valorizing AV rind waste into value-added products through sustainable waste management would contribute to the circular economy. In this respect, Cheng et al. mentioned that AV rind allows the obtaining of nanofiber-reinforced films due to the presence of a high amount of α-cellulose. However, the processing of AV rinds into new materials and their potential uses has not been sufficiently studied [33].

In this work, AV rind waste biomass obtained from Hidalgo, a state in the center of Mexico, is processed and studied to obtain crystalline nanocellulose (CNC) through alkaline and acid pretreatments and two types of hydrolyses (citric and sulfuric acids) approaches. The effects of the reaction on the functional groups, hydrogen bonding type, polymorph contribution, crystallinity, morphology, particle size, and metal content are discussed to determine the final applicability and provide a basis for the optimal methods for valorizing AV into reinforcement agents.

## 2. Materials and Methods

### 2.1. Crystalline Nanocellulose (CNC) Extraction

#### 2.1.1. Cellulose Extraction from AV Rinds

AV leaves were collected from Hidalgo, México (20.112534828824856, −98.84498557997675) and chopped; the thorns were discarded. The gel on the AV rinds was removed with distilled boiling water for 1.5 h, ground in a blender, and sieved to remove the excess water. The AV biomass was placed in aluminum trays and dried in an oven at 70 °C overnight until the fibers were dried. It was then milled using a laboratory mill CT 293 Cyclotec™ (FOSS, Hilleroed, Denmark) using a 0.3 mm mesh.

The AV powder was subjected to the Soxhlet extraction method to remove the waxes, pigments, and pectin. This process evaporated a 1:1 ratio of a mixture of deionized water and absolute ethanol (99.9% purity, J.T. Baker, Phillipsburg, NJ, USA) in a balloon flask to condense it at the top of the system and deposit it into a siphon. This occurs at the saturation temperature of the liquid where the AV biomass is located. A cellulose thimble (4.0 × 12.3 cm, Whatman International Ltd., Buckinghamshire, UK) was used to permeate the pigments and pectin in the siphon. A total of 4 cycles of 2 h were employed. The AV biomass was then left to cool at room temperature and dried at 70 °C.

An amount of 40 g of the AV biomass was added to a round-bottom flask with 400 mL of a 0.1 M sodium hydroxide (NaOH, J. T. Baker, ≥98.0%) solution, which was closed with a silicone rubber septum to remove hemicellulose. A pin was inserted to prevent high internal pressure. The flask was heated to 100 °C, and the solution was mixed using a magnetic stirrer for 2 h. The AV biomass was filtered using medium-pore paper and neutralized (pH = 7) using hot distilled water. The AV biomass was placed in an oven to dry at 70 °C overnight.

The bleaching process to remove lignin consisted of weighing 40 g of dry AV biomass and adding 350 mL of 3% (*wt*/*v*) of sodium chlorite (NaClO_2_, 80% purity, Sigma Aldrich, Darmstadt, Germany) in a balloon flask with a magnetic stirrer. An amount of 7 mL of acetic acid was added and heated at 80 °C for 2 h. The solution was filtered, washed, and neutralized using hot distilled water until a pH of 7 was reached. The sample was dried at 90 °C. This process was repeated.

#### 2.1.2. CNC Extraction from AV Rinds

Acid hydrolysis was performed using two approaches to remove the amorphous cellulose region. An amount of 10 g each of bleached AV biomass was prepared, adding 60 mL of 64% (*v*/*v*) sulfuric acid (H_2_SO_4_, 93–98%, Fermont, Monterrey, Mexico). The same procedure was performed using 60 mL of 64% (*wt.*/*v*) of citric acid (Sigma Aldrich, ≥99.5%). Each procedure was carried out under magnetic stirring at 50 °C for 30, 60, and 90 min. An amount of 60 mL of cold water was added to stop the reaction at the end of the hydrolysis time. Samples were inserted into dialysis bags [avg. flat width 43 mm (1.7 in), Sigma Aldrich)] and submerged in deionized water. The water was replaced every 24 h until the neutral pH was reached. The CNCs were obtained at the end of this process. In order to characterize it, CNCs were dried at 70 °C. The samples were named CNC_SA30_, CNC_SA60_, CNC_SA90_, and CNC_CA30_, CNC_CA60_, and CNC_CA90_, where SA and CA mean sulfuric acid and citric acid, respectively, and the 30, 60, and 90 mean the minutes of reaction during hydrolysis.

#### 2.1.3. Structural, Thermal, and Morphological Characterization

Functional group analysis of AV rinds, cellulose, CNC_SA_, and CNC_CA_ samples were analyzed by Fourier Transform Infrared Spectroscopy (FTIR) using a Spectrum 100 Spectrometer (PerkinElmer, Shelton, CT, USA) with an attenuated total reflectance (ATR) accessory of zinc selenide (ZnSe). Transmittance (%) measurements were performed in a range from 4000 to 500 cm^−1^ with a total of 10 scans for analysis. Deconvolution of OH signals in the 3800–3000 cm^−1^ region was performed to evaluate hydrogen bonding contributions using OriginPro (ver. 10.1.0.178, 2024) software considering the Gaussian distribution function (fitting correlation ≥ 0.99). The content percentage for each type of hydrogen contribution was estimated by comparing the total integrated area of the main peak (OH contribution) with respect to the area of each deconvoluted signal, as previously reported [34].

Binding energy [35] was calculated according to Equation (1):

(1) $$E_{H}=\frac{1}{k}\left[\frac{v_{0}-v}{v_{0}}\right]$$

where $v_{0}$ is the wavenumber of free OH signals at 3650 cm^−1^, v is wavenumber of bonded-OH signals, and k is a constant (2.625 × 10^2^ kJ).

Bond lengths were also calculated according to the Sederholm [36] Equation (2):

(2) $$\Delta v=4.43\times 10^{3}\;\left(2.84-R\right)$$

where R is the hydrogen bonding distance, Δv is the $v_{0}-v$, $v_{0}$ is the OH stretching wavenumber at 3600 cm^−1^, and v is the stretching wavenumber detected in the sample [35].

The crystalline structure of cellulose and CNC_SA_ and CNC_CA_ were analyzed via the X-ray diffraction (XRD) technique in a Bruker D8 Advance diffractometer (Bruker, Billerica, MA, USA) in a range from 10 to 40° (2θ) using Cu Kα radiation and a step size of 21.4 s/step. The crystallinity percentage (Cr%) was calculated through the peak area method considering Equation (3):

(3) $$C_{r}\%=\frac{Area_{Cr}}{Area_{sample}}=\left[\frac{\int_{2\theta_{1}}^{2\theta_{2}}I_{Cr}d2\theta }{\int_{2\theta_{1}}^{2\theta_{2}}I_{sample}d2\theta }\right]100$$

$Area_{Cr}$ is the area of crystalline peaks, $Area_{sample}$ is the area of the sample, $I_{Cr}$ relates to integral sum of crystalline peaks intensities, and $I_{sample}$ is the sum of intensities of all diffraction space [37].

The polymorph contribution content (%) for each pretreated sample was analyzed from each XRD pattern using the Match! Software (version 4.0, build 295), considering the crystallographic information (CIF) file for the crystalline structure of Iα (triclinic), Iβ (monoclinic), and II (monoclinic); the software employs the reference intensity ratio method for the estimations. This methodology has been employed by M. L. M. Rocha et al. [38].

Thermogravimetric analysis (TGA) of AV rinds, after being pretreated with soxhlet, NaOH, and NaClO_2_/CH_3_COOH, to obtain cellulose, CNC_SA_, and CNC_CA_, was analyzed to determine the decomposition temperatures (Td) in a thermal analyzer SDT-Q600 (TA Instrument Inc., New Castle, DE, USA) from room temperature to 800 °C at 10 °C/min, using aluminum crucibles under argon atmosphere (100 mL min^−1^).

The morphology of selected CNC_SA_ and CNC_CA_ was carried out via Scanning Electron Microscopy (SEM), using a field emission microscope JSM-6701F (JEOL, Tokyo, Japan) of an ultrahigh resolution at 5 kV.

Dynamic light scattering (DLS) was analyzed in a Litesizer 500 analyzer (Anton Paar GmbH, Graz, Austria) in a quartz cell at 22 °C using 50 runs. An amount of 1 mg of selected CNC_SA30_ and CNC_CA90_ samples were individually dispersed in 10 mL of HPCL grade water and sonicated for 10 min.

Selected CNC_SA30_ and CNC_CA90_ samples were digested in nitric acid in autoclave. Elemental determination was performed in an ICP-OES, OPTIMA 2100DV model (PerkinElmer, Shelton CT, USA). A total of 17 elements were analyzed.

## 3. Results and Discussion

### 3.1. Monitoring of Functional Groups and Hydrogen Bonding Interactions Through AV Rinds Pretreatments to Obtain CNCs

Functional group monitoring through FT-IR analysis was performed to evaluate those more susceptible to removal and associate them with extractives, hemicellulose, and lignin. This step is key to verifying the effectiveness of the method.

**Figure 1:**
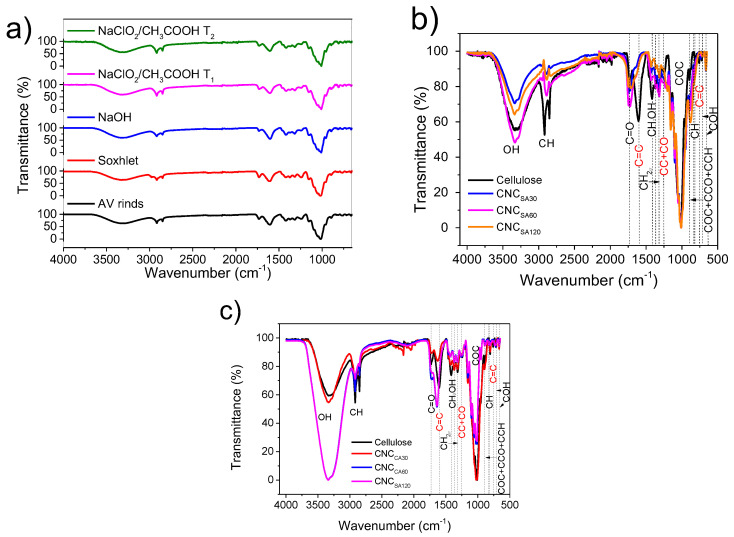
FTIR spectra of (**a**) AV pretreatment to remove extractives, hemicellulose, and lignin, (**b**) CNC_SA_ and (**c**) CNC_CA_.

The Aloe Vera rinds spectrum contains the following functional groups which were identified in the FT-IR spectrum (Figure 1a): the stretching of OH group due to intermolecular bonding with hydrogen in cellulose; hemicellulose and lignin (3337 cm^−1^); asymmetric stretching of CH in CH_2_, CH_3_, and CH groups (2916 cm^−1^); symmetric stretching of CH in CH_2_ groups (2850 cm^−1^); stretching of C=O carbonyls (1732 cm^−1^); stretching of aromatic skeletal of C=C lignin groups (1600 cm^−1^) [39]; in-plane bending of CH_2_ and CH_2_-OH groups (1420 cm^−1^); bending vibration of CH_2_-OH group (1372 cm^−1^); CH_2_ wagging of crystalline region in cellulose (1317 cm^−1^); stretching of CC + CO in condensed and etherified guaiacyl in lignin (1244 cm^−1^); asymmetric stretching of the COC group (1160 cm^−1^); symmetric stretching of the COC group (1018 cm^−1^); deformation of COC, CCO, and CCH stretching (890 cm^−1^); CH out-of-plane vibration (835 cm^−1^); CH vibration in glucomannan and galactomannan rings (813 cm^−1^); vibrational modes of paraben in lignin (762 cm^−1^); out-of-plane bending of C-OH (720 cm^−1^); out-of-plane bending of C-OH (670 cm^−1^); and out-of-plane bending of C-OH (665 cm^−1^).

The presence of C=O groups with higher intensity than the initial biomass was observed in the spectrum of Figure 1 (Soxhlet) because carbonyls were more easily detected using the tip of the equipment now that the extractives had been removed. The groups related to CH overlapped with those of cellulose, lignin, and hemicellulose decreased in intensity after this pretreatment (1500–1130 cm^−1^). Also, OH groups corresponding to the ethanol employed at 2980 cm^−1^ were still present [40]. Sodium hydroxide had the ability of ester and ether bonds cleavages in the biomass, dissociating it into Na and OH ions to promote hydrolysis [41]. For this reason, the spectrum (NaOH) displayed high-intensity OH groups due to the generated hydrolysis forming more of these groups. A reduction of C=O and CH_2_-OH groups due to the hemicellulose removal [42] and solubilization was detected, as well as the signals corresponding to lignin (CC + CO) since NaOH also penetrates into this aromatic polymer removing it [43].

With respect to the sodium chlorite/acetic acid pretreatment (NaClO_2_/CH_3_COOH T_1_ and T_2_), changes were observed in the bands located at 1732 cm^−1^ and 1640 cm^−1^ corresponding to the C=O and C=C groups, which are present in lignin. The bleaching pretreatment was carried out with the objective of removing the lignin, so the reduction of the intensity of functional groups, mainly the C=C (1600 cm^−1^) and CC + CO in lignin (1244 cm^−1^), corroborated its efficiency in removal in Aloe Vera. There was no significant change in the bands corresponding to cellulose between the first and second bleaching, so we can infer that sodium chlorite/acetic acid does not structurally modify cellulose.

Figure 1b shows the spectra of comparison of cellulose hydrolyzed with sulfuric acid (SA) for CNC (a) 30 min (CNC_SA30_), (b) 60 min (CNC_SA60_), and (c) 90 min (CNC_SA90_). The C=O signal of the CNC_SA90_ spectrum shifts into a high wavenumber (Δ = 10 cm^−1^), typical of a hydrolysis reaction, derived from the different molecular weights of cellulose chains [44]. This signal corresponds to ester groups in CNC [45]. There is also the presence of carbonyl groups due to the oxidation of cellulose appearing at 1720 cm^−1^ [46]. CNC_SA60_ is that which presents high-intensity bands. Hydrolysis of all three systems led to the total disappearance of C=C groups corresponding to lignin, demonstrating the efficacy of the sulfuric acid to also remove lignin remnants; this feature was also observed at 1244 cm^−1^ (CC + CO) of the lignin groups [47]. Another signal disappearing from bleached cellulose corresponds to 1420 cm^−1^, previously reported to be related to cellulose I [48]. This fact could be related to the transition from cellulose I to II. A low-intensity band of CH_2_-OH group of cellulose relates to amorphous cellulose I (1372 cm^−1^) [12], which is totally removed after acid hydrolysis with SA. The peak appearing in all CNC_SA_ samples at 1365 cm^−1^ corresponds to the breakage of inter- and intrahydrogen bonds [49]. A new arising signal corresponding to the (SO_4_)^−3^ attack of C_6_ of pyranose ring at 1230 cm^−1^ was detected [50]. There was a new peak at 1227 cm^−1^ related to crystalline cellulose [51]. This feature was not observed in the CNC_SA90_ sample, probably due to the high hydrolysis time. The signal c.a. 1160 cm^−1^ became defined and high intensity as the hydrolysis time increased from 30 to 90 min and was related to the increase of crystalline areas of cellulose [51]. Another aspect related to crystallinity was the signal of CH groups at 955 cm^−1^ disappearing after 30 min in CNC_SA30_ and CNC_SA90_ samples [52]. An interesting feature was observed at 895 cm^−1^, confirming the transition from cellulose I to II [52] through the increase of intensity and the shifting of the signal during 90 min of hydrolysis. Due to its removal through time, the 830 cm^−1^ band of amorphous cellulose is well-perceived in CNC_SA30_ and CNC_SA60_ samples but not in CNC_SA90_ [16].

The spectra of hydrolyzed with citric acid (CA) samples in Figure 1c show similar functional groups with small differences in the carbonyl regions, which were reduced in intensity with hydrolysis due to the reaction with citric acid molecules and ester groups [45,53]. This signal was less intense than cellulose due to the ester of carboxylic acid in CNC_CA30_ [54]. There was a shift to a low wavenumber (Δ = 14 cm^−1^) after hydrolysis for 60 min. CNC_CA60_ and CNC_CA90_ were also of low intensity, but the last one exhibited evidence of oxidation due to the length of hydrolysis. Esterification of carboxylic acid is advantageous since hydrogen bonds are reduced, improving repulsion [54]. In this sense, intra- and intermolecular hydrogen bonds are essential in forming the crystalline packaging and determining final applications.

Figure S1 in Supplementary Material shows the OH region deconvolution from the spectra of Figure 1. The contribution of O(3)···H···O(5) and O(2)···H···O(6) interactions correspond to intramolecular and O(6)···H···O(3) to intermolecular hydrogen bonding, respectively. The first contributes to axial stiffness, and the second maintains the structural integrity [55]. Aloe Vera rinds are characterized by having both inter (37.10%) and intra (62.89%) bonding in which the first is completely loosened after removing extractives through Soxhlet, NaOH, and NaClO_2_/CH_3_COOH pretreatments (Table 1). Those pretreatments weakened the intermolecular forces holding the AV biomass molecules [56]. However, it was noticed that intermolecular bonds can be again formed when extracted cellulose is obtained by employing the second NaClO_2_/CH_3_COOH pretreatments; cellulose is characterized to be 31.95% and 68.04% of O(3)···H···O(5) (intra) and O(6)···H···O(3) (inter) interactions, respectively. After hydrolysis with sulfuric acid, intermolecular bonding was reduced to 33–36% (CNC_SA30_ and CNC_SA60_). Sulfuric acid can break those interactions by penetrating the crystals [20]. There is an increase in intermolecular interactions by up to 52.50% due to the presence of more hydroxyl units derived from the longer period of hydrolysis [57]. Y. Sun et al. have shown a similar result, indicating a decrease and further increase due to cellulose bundling together during hydrolysis [58].

In contrast, the behavior of disrupting interbonding was not detected for the citric acid group’s hydrolysis, since the percentages were maintained after 30 min of hydrolysis (CNC_CA30_, 67.23%), decreasing up to 46.15% and 59.31% in CNC_CA60_ and CNC_CA90_. In this case, the interaction during the hydrolysis with citric acid supplemented those interbondings, as observed in other systems such as starch [59]. Therefore, it is essential to highlight that more studies must be carried out to analyze the type of contributions made by hydrogen bondings to better understand, for example, whether this is due to alpha or beta type I or II cellulose.

### 3.2. Crystalline Structure Determination and Polymorphism After Pretreatments

**Figure 2:**
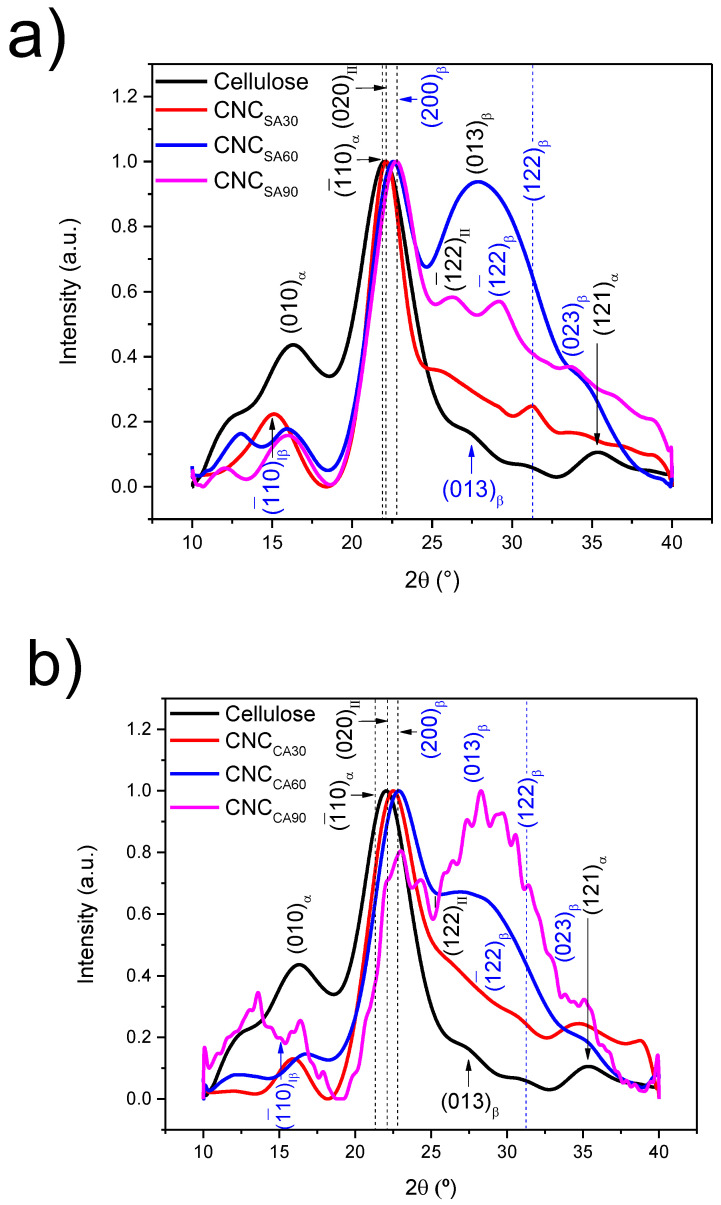
XRD patterns of cellulose and (**a**) CNC_SA_ and (**b**) CNC_CA_.

In the crystalline zone of cellulose, the polymers physically interact through hydrogen, Van der Waals, and hydrophobic contributions; they are held together, but after pretreatment employing chemical or physical methods, the obtained polymorphs in cellulose can vary [60,61]. For this reason, XRD was employed to evaluate the crystal type present after both hydrolysis types. Figure 2 displays the XRD patterns, and Table 2 shows the polymorph contribution content and crystallinity percentage (C_r%_).

Extracted-cellulose from AV rinds displayed the $\left(010\right),\;\left(\bar{1}10\right),\;\left(121\right)$, and $\left(013\right)$, and $\left(122\right)$, crystallographic planes, corresponding to the Iα triclinic and Iβ monoclinic structures, respectively. These polymorphs were found in 59.6% and 40.4%, respectively, with minimal amounts of monoclinic type II cellulose (5.1%) contributing to 25.12% of the crystallinity. Compared to the cellulose sample, hydrolysis with sulfuric acid produced a shift towards a high 2θ value (Δ = 0.14°) in the XRD pattern after 30 min (CNC_SA30_) at the $\left(\bar{1}10\right)$ plane of the cellulose sample; this was the most intense signal, due to the observed transformation to Iβ allomorph and the appearance of type II cellulose—plane (020). This feature is consistent with the disintegration of 23.2% of the triclinic structure, leaving a more thermodynamically stable structure—the Iβ [62]. Other important observations were the appearance of the $\left(\bar{1}10\right)$ and (122) planes of Iβ and a significant increase of crystallinity (70.85%) due to the cellulose’s amorphous removal. The phases content was 26.2% (Iα), 28.1% (Iβ), and 45.7% (II). A longer hydrolysis time in the XRD pattern did not significantly impact the C_r%_ (73.70%), but it did the type II content. The XRD of CNC_SA60_ indicated a similar result at the main overlaps ($\bar{1}10$) and (200) peaks of Iα and II, with a shift of 0.54° displaying a broad (103) signal Iβ phase. The phase contribution of Iα, Iβ, and II was 8.1, 35.0, and 56.9%, respectively, i.e., a 41.3% transformation into Iβ or II polymorphs compared to CNC_SA30_. Finally, CNC_SA90_ was very similar in composition to CNC_SA60_, having lower C_r%_ (62.75%) than CNC_SA30_ and CNC_SA60_, probably due to the cellulose decomposition, but, in this case, of the crystalline regions. This was previously reported in other works to be due to the longer reaction time [63]. In fact, the reason behind the crystallinity reduction is due to the intramolecular hydrogen bonding content detected by FTIR deconvolution which decreases at a long hydrolysis time (CNC_SA90_). S. Singh et al. have also detected this behavior in cellulose nanocrystals from Cajanus cajan (pigeon peas) [55].

In comparison to the broad (103) plane in CNC_SA60_, the XRD pattern displayed better-defined signals belonging to ($\bar{1}22$) of II and ($\bar{1}22$) of the Iβ phases. A longer hydrolysis time in CNC_SA90_ had no effect on the polymorph content; 14.1%, 32.7%, and 53.2%, respectively. However, it is notable that a shift in the ($\bar{1}10$) plane with d= 5.95 Å of beta polymorph into high 2θ value occurred, which has been reported to be related to changes in conformations of intermolecular hydrogen bonds, as detected previously by Y. Takahara et al. [64].

These results are of great significance for valorizing AV rinds into CNC, since there is the knowledge concerning its structural properties through XRD is poor. D. Jangam Seshagiri et al. extracted CNC from AV rinds to develop a film with poly(vinyl alcohol) using 45 min at 45 °C, having no report about C_r%_ and polymorph identification [65], essential parameters in analyzing the final properties of composites. M. A. Guancha-Chalapud et al. extracted CNC from AV rind cuticles to develop a hydrogel, but only the morphological, structural, and thermal properties were analyzed [66]. On the contrary, S. Cheng et al., extracted nanofibers from AV rinds through a chemical–mechanical process, obtaining no significant change in the crystallinity compared to the raw material—from 59% to 66% [33].

For citric acid hydrolysis, it is notable that the XRD pattern of CNC_CA30_ and CNC_CA60_ displays similar behavior to that of samples processed with sulfuric acid. Especially the shifting of the more intense and primary signal into high 2θ (°), which was also detected with sulfuric acid pretreatment. As the hydrolysis time increases from 30 to 60 min, the difference in shifting increases with a Δ = 0.48 and 0.76, respectively. Those differences in value are even higher than those observed in the CNC_SA_ samples. In both, the presence of Iα, Iβ, and II was found to be very similar in content: 14.4%, 32.6%, and 53%, respectively, for CNC_SA30_ and 11.7%, 33.7%, and 54.7%, respectively, for CNC_SA60_. Crystallinity increased up to 63.53% and 64.38%. A different XRD pattern was observed for CNC_SA90_ due to the presence of the citric acid molecule (PDF # 96-500-0064) by the esterification previously detected via FTIR analysis. Some authors have compared the XRD pattern when processing citric acid either to evaluate removal or addition with other molecules [67,68]. The effect of this esterification on the crystallinity or polymorph content indicates a slight improvement of crystallinity c.a. 13–14% (C_r%_ = 63–64%) when employing 90 min and having a higher amount of type II cellulose compared to 30 and 60 min. C_r%_ was also found to be dependent on the intramolecular hydrogen content detected in the FT−IR analysis.

Therefore, the structural analysis indicated that 30 min of acid hydrolysis with sulfuric acid and 90 min with citric acid are enough to obtain CNCs with the highest crystallinity percentage of extracted cellulose from AV rinds, which are composed of a mixture of polymorphs but are predominant in type II cellulose.

### 3.3. Thermal Stability of AV Rinds After Pretreatments and CNCs

**Figure 3:**
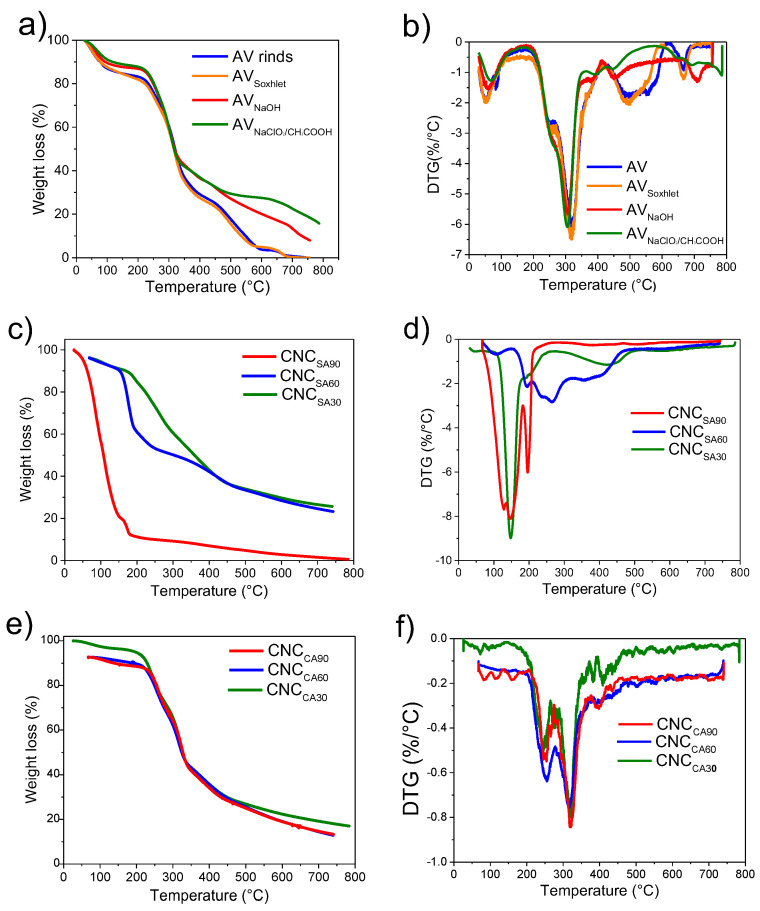
TGA and DTG thermograms curves of (**a**,**b**) AV after each step of pretreatment, (**c**,**d**) CNC_SA_ and (**e**,**f**) CNC_CA_.

Figure 3 shows TGA and derivative thermogravimetric (DTG) thermograms for (a,b) AV rinds and their pretreatments to obtain cellulose, (c,d) CNC_SA_, and (e,f) CNC_CA_. Table 3 summarizes the onset temperatures, final temperatures, and degradation peak positions obtained from the DTG curves.

The TGA curves showed three steps. The first, between 30 and 229 °C, is attributed to absorbed moisture on the surfaces of these materials, including chemisorbed and intermolecularly H-bonded water. Interestingly, AV rinds and all the samples of AV after pretreatments presented this first stage in a very similar range from 30 to 210, 213, 212, and 206 °C, respectively. However, the CNC obtained after hydrolysis treatments showed significant changes; samples treated using sulfuric acid needed high temperatures for moisture loss (CNC_SA30_: 229, CNC_SA60_: 211, and CNC_SA90_: 219 °C), showing low mass loss compared with the CA treatment. For example, the CNC_SA60_ sample had a mass loss of 7.69 wt%, showing an increase in the crystalline percentage of the samples treated with sulfuric acid. Water molecules cannot interact with the crystalline regions of CNC_SA_, and the crystalline regions require more thermal energy to liberate the moisture absorbed. The samples treated using CA needed low temperatures for moisture loss (153, 126, and 148 °C) and showed a high moisture content; the sample of CNC_CA90_ had a mass loss of 78.48 wt% during this first step (I). This behavior in the samples treated using CA can be attributed to a less severe hydrolysis reaction with a low crystallinity percentage; amorphous regions absorb more water molecules. On the other hand, using CA, an increase of hydrophilic functional groups (COOH) on the surface of CNC provokes a reduction of the energy needed to liberate the water because the hydrogen bond interaction is easy to break [69]. Furthermore, it is well known that the carboxylic acid groups (COOH) of citric acid can react with the hydroxyl groups (OH) of cellulose, forming ester bonds (R-COO-R′) through an esterification reaction that occurs around 150 °C, which liberate water as a secondary product of the reaction forming crosslinking networks [59,70].

The second step of the TGA curve (II) is attributed to the degradation of the materials. This stage is between 126 and 449 °C. Thermal degradation of the AV rinds and samples after pretreatments was between 382 to 390 °C. Thermal degradation temperatures of the samples after acid hydrolysis using SA for 30, 60, and 90 min were lower than with CA, with values of 333, 326, and 328 °C, respectively. This reduction of the degradation temperature of the samples treated using SA could be related to the replacement of the OH groups of cellulose by acid sulfate groups (O-SO_3_-H), decreasing the activation energy for the degradation of CNC, therefore, making them less resistant to pyrolysis degradation [54].

The samples treated by acid hydrolysis using CA for 30, 60, and 120 min had high degradation temperature values of 444, 449, and 191 °C, compared with samples of acid hydrolysis using SA. This increase of the thermal stability of CNC obtained using CA can be attributed to the esterification reaction of the cellulose with citric acid in noncontrolled reaction conditions that produces a crosslinking structure [71]. The maximum degradation temperature observed by DTG for these samples obtained by CA hydrolysis decreased as the reaction time increased (437, 405, and 170 °C), probably due to a greater insertion of COOH groups on the CNC surface and, therefore, a more significant occurrence of crosslinking events at elevated temperatures [72].

The same tendency of results can be observed in the third step of the degradation process (III). The high temperature of degradation is observed in CNCs obtained using CA with a final degradation temperature of 700 °C for the three times treatments (30, 60, and 90 min) and the low temperature of degradation was observed for the SA for 30, 60, and 90 min with values of 450, 489, and 472 °C, respectively. It can be concluded that the hydrolysis process determines the thermal properties of the CNC.

### 3.4. Morphological Analysis of CNC_SA_ and CNC_CA_

**Figure 4:**
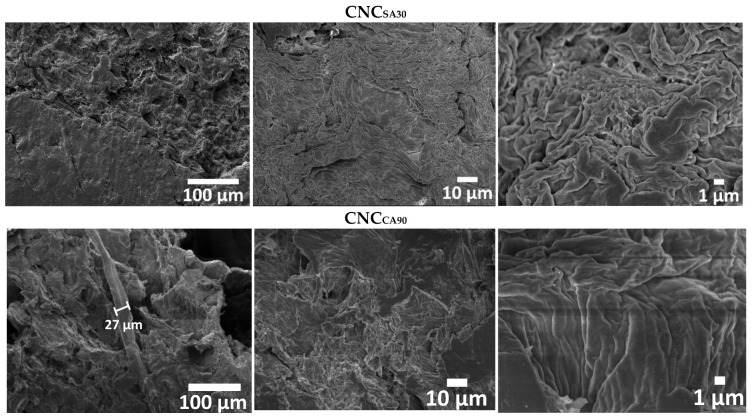
SEM micrograph of CNC_SA30_ and CNC_CA90_.

The final morphology of selected CNC_SA30_ and CNC_CA90_ is shown in Figure 4. The surface of CNC_SA30_ displays the internal morphology of cellulose with a smooth surface and some porous. There was no evidence of single fibers because the samples were analyzed in a compacted-film shape. The CNC_CA90_ sample’s morphology was very similar but slightly rough compared to CNC_SA90_ due to the different types of hydrolysis. This sample displays a ribbon single-fiber c.a. 27 µm of diameter. These morphologies have been reported in pretreated rice straw as porous and highly accessible to enzymes [29]. R. Gunasekaran et al. reported an increase in roughness and crack formation after pretreated aloe vera leaf rinds due to distortions on the cell wall surfaces [73].

### 3.5. Particle Size Estimations of Selected CNC_SA30_ and CNC_CA90_

**Figure 5:**
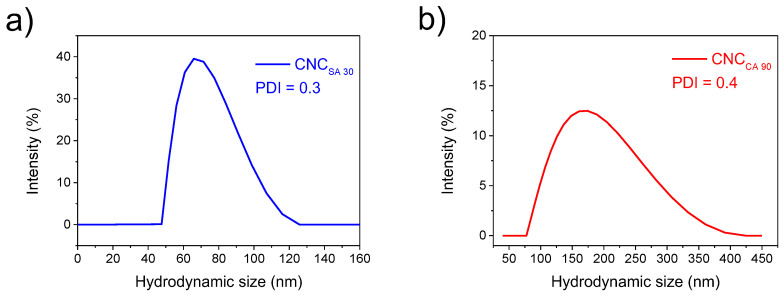
DLS graph corresponding to the particle size distribution of selected (**a**) CNC_SA30_ and (**b**) CNC_CA90_.

The particle size estimation of CNC is very important in the plastic field since the nanoparticles typically exist as aggregates of primary particles. The final properties depend on the interaction with the surrounding materials because rarely are the nanoparticles employed by themselves. Instead, they typically are dispersed and combined with other materials through different processing methods [74]. Cellulose-based particles have increased stiffness and strength properties in composites at low contents [63,75]. In this work, CNC_SA30_ was found in the 45–120 nm range, whereas CNC_CA90_ in the 75–400 nm (Figure 5). As observed, the size distribution of the particles hydrolyzed with citric acid is higher than with sulfuric acid. The polydispersity index (PDI) was found within the acceptance level according to Y. Sun et al. [76]. The low particle size is due to sulfuric being a stronger acid than citric in destroying the amorphous zone of cellulose [77]. In this case, particle size depends on the strong or weak acid, concentration, temperature, and hydrolysis time [78]. The reason behind the broader and higher particle size in CNC_CA90_ than CNC_SA30_ is related to size since an esterification reaction was detected introducing COO^-^groups in the CNC_CA90_ molecule. This feature has been reported as a larger contact surface area due to the esterification reaction of the citric acid [69]. Other studies in processing cellulose nanocrystals from bleached bagasse pulp have reported small sizes of 20–30 nm and 30–60 nm [65], which are related to the present work in the esterification of cellulose when using citric acid. Compared to other AV rinds valorization, E. Triantafyllou et al. apparently extracted cellulose nanocrystals to reinforce poly(vinyl alcohol) (PVA), but no knowledge about particle size was reported [1]. This also happens in a study by A. N. Balaji et al., where they reported on cellulosic fiber from Saharan Aloe Vera cactus leaves [61]. Other Aloe Vera, Saharan types, extracted through acid hydrolysis, result in lower sizes c.a. 10–14 nm than CNC_SA30_ and CNC_CA90_ [66]. Whereas S. Cheng et al. reported sizes smaller than 100 µm having diameters under 20 nm and others between 20 and 100 nm employing alkaline, acid, and mechanical pretreatments [33].

### 3.6. Elemental Analysis of AV Rinds Biomass and Selected CNC_SA30_ and CNC_CA90_

Lignocellulosic biomass includes inorganic matter such as macronutrients and micronutrients. Macro- and micronutrients are needed for plant growth in high and low concentrations. For example, nitrogen (N), potassium (K), magnesium (Mg), phosphorous (P), calcium (Ca), and sulfur (S) are examples of macronutrients, whereas copper (Cu), manganese (Mn), zinc (Zn), iron (Fe), boron (B), molybdenum (Mo), and chlorine (Cl) are micronutrients. Other elements include sodium (Na), vanadium (V), nickel (Ni), cobalt (Co), aluminum (A), silicon (Si), and selenium (Se). The specific inorganic matter depends on the type of biomass [79]. Additionally, AV rinds can contain elevated amounts of inorganic elements because the AV plant is prone to contamination from absorbing heavy metals from the air, water, and soil. Additional sources of these elements include plant protection agents, industrial activities, rainfall, and atmospheric dust [80].

Table 4 displays the elemental composition of AV rind biomass and selected CNC_SA30_ and CNC_CA90_. In this case, Ca (1400 mg kg^−1^) and Mg (2350 mg kg^−1^) are the main inorganic elements in the AV rind biomass. Transition metals detected include Cr (0.726 mg kg^−1^), Mn (3.23 mg kg^−1^), Fe (31.8 mg kg^−1^), Ni (15.4 mg kg^−1^), and Cu (7.1 mg kg^−1^). Furthermore, post-transition metals, such as Al (44.54 mg kg^−1^), Cd (0.438 mg kg^−1^), Pb (4.87 mg kg^−1^), and Zn (12.29 mg kg^−1^), along with non-metals, metalloids, and alkali metals, such as Se (5.62 mg kg^−1^), B (6.64 mg kg^−1^), and Li (0.312 mg kg^−1^), were also detected. As and Mo were not detected in this analysis. In a recent study, Kalderis et al. reported the presence of these transition metals in AV leaves from Chania, Greece, with concentrations generally lower than those observed in this study, except for Cr. Elements such as Ni, As, Cd, Cr, and Pb are considered hazardous due to their high toxicity to living organisms and the environment [68].

In the case of elemental concentrations for CNC_SA30_, it is clear that the effect of alkaline/acid pretreatments with the acid hydrolysis, with sulfuric acid, leads to a decrease in almost all elements initially present in AV rind biomass. For instance, Ca (433 mg·kg^−1^), Mg (2091 mg·kg^−1^), Fe (15.96 mg·kg^−1^), B (4.64 mg·kg^−1^), Zn (10.32 mg·kg^−1^), Cu (6.65 mg·kg^−1^), Mn (2.4 mg·kg^−1^), Ni (12.7 mg·kg^−1^), Al (42.7 mg·kg^−1^), and Se (3.03 mg·kg^−1^) were all reduced. This observation supports previous findings on the effects of acid, alkaline, and hydrothermal pretreatments of different biomass sources, where a reduction in elemental concentrations was similarly noted [67].

Regarding CNC_CA90_, an increase in the elemental concentrations was observed, especially in the case of Ca (24,157 mg·kg^−1^), Cr (2.03 mg·kg^−1^), Ni (23.95 mg·kg^−1^), Mg (23,746 mg·kg^−1^), and Pb (12.27 mg·kg^−1^). However, these values are below the limits set by the EU for fertilizing product categories (120 mg·kg^−1^ for Pb, 2 mg·kg^−1^ for Cr, and 50 mg·kg^−1^ for Ni) [70]. CNC_CA90_ holds a higher amount of (Ca 24,157 mg/kg) and Mg (23,746 mg/kg) compared to native AV rind biomass (1400 mg/kg and 2350 mg/kg, respectively) due to the employed chemicals, which were technical grade and contained these metals as impurities. Notably, the presence of esterification with citric acid leads to apparently a chelation behavior via electrostatic interactions with metals [81,82]. Another observation from this analysis is that lead violates food contact safe concentrations. For this reason, CNC_CA90_ is not suitable for processing food products. However, it is necessary to establish several methodologies to remove this metal, such as those employed in reusing adsorbent-based cellulose materials; using 0.1 M solution of nitric acid [83]. However, it is essential to clarify that future studies must be performed to corroborate this lead elimination method.

## 4. Conclusions

Hydrogen bonding contributions were successfully explored after obtaining cellulose and CNCs from AV rind biomass. The pretreatments reduced the structure’s stiffness due to the removal of extractives such as pigments, hemicellulose, and lignin. Intermolecular hydrogen bondings govern cellulose from AV rinds, which are reduced with strong sulfuric acid to obtain CNC_SA_. Hydrolysis with citric acid maintained the intermolecular interactions in CNC_CA_. Both hydrolyses led to the transformation of type I into II polymorphs with high crystallinity compared to extracted cellulose from AV rinds. CNC_SA_ and CNC_CA_ optimal samples were found at the nanometric size, especially those pretreated with sulfuric acid. Sulfuric acid pretreatment led to reduced micro- and macronutrients as well as toxic elements initially present in the AV rind biomass, whereas citric acid led to a chelation behavior with the detected elements. Optimal CNC_SA30_ and CNC_CA90_ display potential applications to reinforce polymers with no more melting temperatures of 160 °C and 220 °C, respectively, such as poly(lactic acid) (PLA) and poly(ethylene) (PE), respectively.

## Figures and Tables

**Table 1:** Hydrogen bonding data for AV pretreatments.

Sample	Wavenumber	Content	Cellulose Type		Bonding Hydrogen Type		Binding Energy	Bond Lengths
	(cm^−1^)	(%)	I	II	Inter	Intra	(kcal)	(nm)
AV rinds	3426	37.10				✓	2.87	2.88
	3263	62.89	✓		✓		5.56	2.92
Soxhlet	3416	48.16	✓			✓	3.03	2.88
	3256	51.83	✓			✓	5.68	2.91
NaOH	3384	63.02	✓			✓	3.56	2.88
	3218	36.97	✓			✓	6.30	2.92
NaClO_2_/CH_3_COOH/T_2_ (cellulose)	3448	31.95	✓			✓	2.20	2.87
	3278	68.04	✓		✓		5.06	2.91
CNC_SA30_	3555	66.37	✓			✓	3.00	2.88
	3244	33.62	✓		✓		5.70	2.92
CNC_SA60_	3361	63.01	✓			✓	4.05	2.90
	3255	36.98	✓		✓		5.88	2.92
CNC_SA90_	3345	47.49	✓			✓	3.95	2.89
	3264	52.50	✓		✓		5.69	2.92
CNC_CA30_	3443	32.76	✓			✓	4.20	2.90
	3303	67.23	✓		✓		5.55	2.91
CNC_CA60_	3415	53.84	✓			✓	2.58	2.88
	3235	46.15	✓		✓		4.90	2.90
CNC_CA90_	3407	59.31	✓		✓		3.06	2.89
	3219	40.68	✓			✓	6.03	2.92

**Table 2:** XRD composition and crystallinity % of samples.

Sample	Iα	Iβ	II	C_r%_
Cellulose	49.4	45.4	5.1	25.12%
CNC_SA30_	26.2	28.1	45.7	70.85
CNC_SA60_	8.1	35	56.9	73.70
CNC_SA90_	14.1	32.7	53.2	62.75
CNC_CA30_	14.4	32.6	53	63.53
CNC_CA60_	11.7	33.7	54.7	64.38
CNC_CA90_	6.7	35.6	57.7	77.73

**Table 3:** Data from TGA and DTG thermograms of AV rinds and their pretreatments to obtain CNC_SA_ and CNC_CA_.

Sample	Td_o_I_	Td_f_I_	%_WL_I_	DTG__I_	Td_o_II_	Td_f_II_	%_WL_II_	DTG__II_	Td_o_III_	Td_f_III_	%_WL_III_	DTG__III_	%_WL_700 °C_
	(°C)	(°C)		(°C)	(°C)	(°C)		(°C)	(°C)	(°C)		(°C)	
AV rinds	30	210	16.86	53.3 83.7	210	390	53.12	250 320	390	600	26.07	550 665	2.39
AV_Soxhlet_	30	213	19.42	53.5	213	386	51.42	251 318	386	571	23.67	493 666	5.19
AV_NaOH_	30	212	13.81	57	212	352	44.19	304.7	352	450	9.79	383 453	18.73
AV_NaClO_2_/CH_3_COOH_	30	206	12.74	67	206	353	45.79	306.9	353	543	13.18	393 448	5.62
CNC_SA30_	30	229	10.54		229	333	43.83	245	333	450	15.95	320	10.3
CNC_SA60_	30	211	7.69		211	326	43.04	235	326	489	20.27	300	11.99
CNC_SA90_	30	219	8.27		219	328	43.56	230	328	472	19.01	310	11.91
CNC_CA30_	30	153	8.3	146	153	444	58.82	437	444	700	11.6		
CNC_CA60_	30	126	8.1	70	126	449	59.3	170 250 405	449	700	13.37		
CNC_CA90_	30	148	78.48	93 113	148	191	8.97	170	191	700	10.6		

Degradation temperatures (Td). Td_o_ onset temperature, Td_f_ final temperature, % weight loss (%WL), and peak decomposition temperatures (DTG) on each decomposition step I, II, and III.

**Table 4:** Elemental analysis for AV rind biomass and selected-CNC_SA30_ and CNC_CA90_ samples.

Metal	AV Rind Biomass	CNC_SA30_	CNC_CA90_
(mg·kg^−1^)			
Al	44.5	42.7	76.1
As	ND	ND	ND
B	6.64	4.64	31.2
Ba	8.98	9.97	44.1
Ca	1400	433	24157
Cd	0.438	0.133	1.06
Cr	0.726	0.217	2.03
Cu	7.1	6.65	16.7
Fe	31.8	15.96	60.3
Li	0.312	0.149	2.28
Mg	2350	2091	23746
Mn	3.23	2.4	15.4
Mo	ND	ND	ND
Ni	15.4	12.7	23.95
Pb	4.87	6.16	12.27
Se	5.62	3.03	5.98
Zn	12.29	10.32	18.55

ND = Not detectable.

## Data Availability

The original contributions presented in this study are included in the article/Supplementary Material. Further inquiries can be directed at the corresponding author.

## Supplementary Materials

The following supporting information can be downloaded at: https://www.mdpi.com/article/10.3390/polym17040553/s1, Figure S1: FTIR spectra corresponding to OH region deconvolution of all pretreatments of Aloe Vera rinds.
