# Supplementary material for: A Strategy Towards the Valorization of Aloe Vera Rinds to Obtain Crystalline Cellulose: Pretreatment Effects and Elemental Analysis
Source: Polymers (Basel). 2025 Feb 19;17(4):553. doi: 10.3390/polym17040553 (PMC11858968; doi:10.3390/polym17040553)
Supplement: Supplementary file 1 [file polymers-17-00553-s001.zip › polymers-3476039-supplementary.pdf]

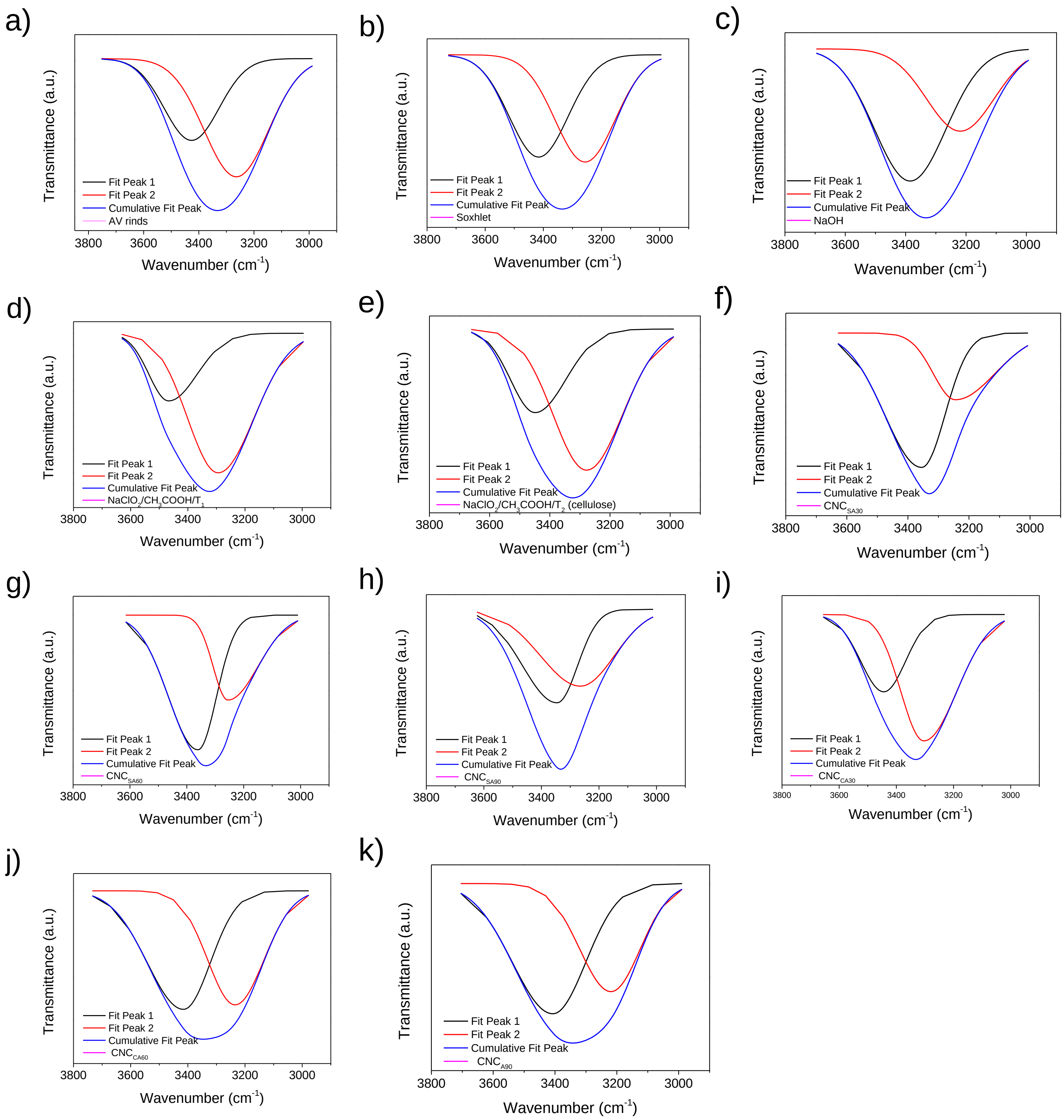

Figure S1. FTIR spectra corresponding to OH region deconvolution of all pretreatments of Aloe Vera rinds.
